# Beta cell response to a mixed meal in nigerian patients with type 2 diabetes

**DOI:** 10.1186/1472-6823-12-11

**Published:** 2012-06-27

**Authors:** Ekenechukwu E Young, Sonny Chinenye, Chioma N Unachukwu

**Affiliations:** 1 Department of Medicine, University of Nigeria Teaching Hospital, Ituku-Ozalla, Enugu, Nigeria; 2 Department of Medicine, University of Port Harcourt Teaching Hospital, Port Harcourt, Nigeria

**Keywords:** Beta cell, Type2 diabetes, Meal stimulation, Glycaemic control

## Abstract

**Background:**

The pathophysiology of type2 diabetes involves both insulin resistance and poor beta cell function. Studies have been done in several populations to assess the relative importance of these mechanisms in individual patients. In our environment studies to assess beta cell function have been done with glucagon stimulation or an oral glucose tolerance test. This study was done to assess the response of the beta cell to a standardized mixed meal and its relationship with glycaemic control in patients with type2 diabetes.

**Methods:**

Ninety patients with type 2 diabetes were recruited into the study. Weight, height, body mass index and waist circumference were measured. Blood samples were analysed for fasting plasma glucose (FPG) and fasting C peptide (FCP) and glycated haemoglobin (HbA1c). Patients were given their usual drugs for management of their diabetes and then served with a standard meal calculated to contain 50 g of carbohydrate, made up of 53 % carbohydrate, 17 % of protein and 30 % of lipids, providing 500 kcal. Blood samples 2 hours after the start of the meal were analysed for postprandial glucose (PPG) and postprandial C peptide (PCP). Fasting (M0) and postprandial beta cell responsiveness (M1) were calculated.

**Results:**

The mean FPG and PPG were 7.51+/− 3.39 mmol/l and 11.02+/−4.03 mmol/l respectively while the mean glycated haemoglobin (HbA1c) was 9.0+/−2.5 %. The mean fasting C peptide was 1.44+/−1.80ug/ml. Many of the patients (56.7 %) had low FCP levels. The mean postprandial C peptide was 4.0+/−2.8 ng/ml. There were significant correlations between M1, HbA1c and PPG (p = 0.015, 0.024, 0.001 respectively) and also between M0, HbA1c, PPG and FPG (p = 0.001, 0.002, 0.001). HbA1c decreased across increasing tertiles of M0 (p < 0.001) and also M1 (p = 0.002). In step-wise linear regression analysis, M0 and M1 significantly predicted HbA1c.

**Conclusions:**

Many of the patients had low C peptide levels with poor beta cell response to the meal. The patients had poor glycaemic control and poor beta cell function. Both fasting and postprandial beta cell responsiveness were significant determinants of blood glucose and glycated haemoglobin levels. It is likely that putting these patients on insulin may have led to better glycaemic control in them.

## Background

In patients with type2 diabetes, poor beta cell function and insulin resistance are the major mechanisms that lead to chronic hyperglycaemia [1]. In the UKPDS, it was demonstrated that at diagnosis beta cell function was reduced by up to 50 % and there was subsequent further deterioration regardless of therapy [2]. It has been postulated that beta cell exhaustion, reduction in beta cell mass and desensitization of beta cells result in beta cell dysfunction [3]. Abnormalities of insulin secretion in persons with overt type 2 diabetes include reduced or absent first-phase responses to intravenous glucose, delayed and blunted secretory responses to ingestion of a mixed meal, alteration in rapid pulses and ultradian oscillation of insulin secretion and an increase in the plasma concentration of pro-insulin [4]. Poor beta cell function occurs to varying degrees and commonly manifests as a poor first phase response to a glucose load [5]. Though beta cell responsiveness has been shown to decrease with increasing duration of diabetes, the rate of deterioration differs in patients. Patients who lose beta cell function early tend to require insulin for glycaemic control at an earlier time from onset of diabetes than others; however beta cell function is not routinely assessed in type2 diabetic patients at diagnosis.

Various models have been used to assess the response of the beta cell to a glucose load, a mixed meal or glucagon stimulation [6]. The use of a meal tolerance test to assess beta cell function is regarded as a more physiological test than the use of a glucose load or glucagon stimulation as it has the benefit of evaluating a typical pancreatic postprandial exposure to glucose and other nutrients and gut and vagal hormones [7].

Some studies have suggested that there are ethnic differences in the degree of beta cell depletion [8,9]. Black South African type 2 diabetic subjects have been found to be more likely than their African-American counterparts to develop depletion of beta-cell function [10]. Basal and stimulated C-peptide levels were also found to be lower in black South Africans compared to South African Indians and whites in both diabetic and non-diabetic populations [10].

The aim of this study was to estimate beta cell response to a standardised meal in Nigerian patients with type2 diabetes and also determine the relationship between their beta cell function and glycaemic control. Other studies on beta cell function done in this environment have been with the use of oral glucose [9], or glucagon stimulation [11].

## Methods

The study was conducted in the diabetes out-patient clinic of the University of Port Harcourt Teaching Hospital, Port Harcourt in the oil-rich Niger-Delta region of Nigeria. Ethical approval for the study was obtained from the hospital ethics committee prior to commencement of the study.

Ninety patients managed for type 2 diabetes who gave written consent were recruited consecutively as they attended the diabetic clinic for follow-up. The patients were assessed with questionnaires to obtain demographic data and those who met the inclusion criteria for the study were asked to report early in the morning on the day of the test after an overnight fast of 8 to 10hours.

### Inclusion criteria

1. Patients earlier classified as having type2 diabetes and on a stable treatment regimen in the last 3 months. Patients were classified as type2 diabetic using clinical criteria. Patients who were above 40 years at diagnosis, did not require insulin for survival, had no features suggestive of secondary diabetes and were not on steroids, oral contraceptive pills or other diabetogenic drugs were presumed to have type2 diabetes and recruited into the study.

2. Patients without any features of metabolic decompensation (i.e evidence of ketosis, hypoglycaemia, advanced heart failure or uraemia) at the time of the test.

3. Patients who gave informed written consent.

### Exclusion criteria

1. Patients with type1 diabetes. These were classified clinically as antibody testing was unavailable. Patients who were less than 40 years at diagnosis and were dependent on insulin for survival were classified as type1 diabetic patients.

On the day of the test, the weight of the patients was measured using a standard scale and recorded to the nearest kg. Height was measured using a stadiometer without shoes or stockings on and recorded to the nearest 0.01 m. Body mass index was calculated with the standard formula; Weight(kg)/height ^2^(m^2^). Waist circumference was measured with a flexible tape with the patient standing erect in light clothing at a point midway between the iliac crest and the lower margin of the rib cage. Blood pressure was measured with a standard mercury sphygmomanometer and the average of two readings taken five minutes apart was recorded.

Fasting venous samples were collected from a peripheral vein for the assessment of fasting plasma glucose and fasting C peptide. After the initial sample, the patients were given a pre-packaged meal which had been formulated to contain 50 g of carbohydrate and 500 kcal and their medications were taken as usual. The mixed meal was made up of 53 % carbohydrate, 17 % of protein and 30 % of lipids. It consisted of a sandwich made with whole wheat bread, fried tomatoes, lettuce and onions and a cup of tea to which had been added 1 % fat milk. The meal was formulated by the hospital dietician. Patients were asked to consume the meal within 15 minutes.

Samples were collected and analysed for glucose and C peptide two hours after the start of the meal. Blood glucose was analysed using the glucose oxidase method while HBA1c was analysed with an in2it machine from Bio Rad Laboratories which is DCCT calibrated and uses the Boronate affinity method. C peptide was analysed using an ELISA based kit (Diagnostic Automation Inc. USA).

The modified formula of Hovorka [12] was used to calculate fasting beta cell responsiveness(M0) and also postprandial beta cell responsiveness(M1). Fasting beta cell responsiveness was taken to be the ability of fasting glucose to stimulate insulin production by the beta cell, while postprandial beta cell responsiveness was defined as the ability of postprandial glucose to stimulate the beta cell.

(1) $$M0=100\times \text{FC peptide}\left(µg/l\right)/FPG\left(\text{mg}/dl\right)\text{.}$$

(2) $$\text{M}1=100\left[PP2-\text{h C peptide}\left(µg/l\right)-\text{FC peptide}\left(µg/l\right)\right]/\left[\text{PPG 2h }\left(mg/dl\right)–\text{FPG }\left(\text{mg}/dl\right)\right]\text{.}$$

### Statistical analysis

Results were analysed with Statistical package for social sciences (SPSS v15). Continuous variables were presented as means ± S.D and categorical variables expressed as frequencies. Chi square test was used to compare categorical variables while the difference between continuous variables was analysed with the students t test or ANOVA. C peptide values, M0 and M1 were log transformed to accommodate the skewing of the variables. In addition, the patients were divided according to their tertiles of M0 and M1 and various clinical characteristics were compared among the three groups. Correlations between duration of diabetes, age, FPG, PPG, HBA1c, M0 and M1 were performed using Pearsons correlation coefficient. Stepwise multiple regression analysis was performed with HbA1c as a dependent variable while variables of age, duration of diabetes, gender, M0 and M1were analyzed as independent variables. A p value of < 0.05 was considered to be statistically significant.

## Results

### Clinical characteristics

The mean age and duration of diabetes were 57.7 ± 10.8 years and 6.77 ± 6.53 years respectively. Oral drugs only were used in 71.1 % of the patients, and this consisted of Metformin alone in 11.1 %, Metformin and Sulphonylurea in 55.6 %, and Sulphonylurea alone in 4.4 %. Insulin (either alone or in combination with metformin) was used in 22.3 % of the patients and only 6.6 % were on diet and lifestyle measures only.

The mean BMI of the patients was 27.54 ± 6.01 kg/m^2^ . Obesity (BMI > 30 kg/m^2^) was present in 16.6 % of the patients while 38.6 % of them were overweight (BMI > 25 kg/m^2^). Hypertension was present in 45.6 % of the patients.

### Glycaemic Control

The mean fasting plasma glucose and 2hour postprandial glucose were 7.51 ± 3.39 mmol/l and 11.02 ± 4.03 mmol/l respectively while the mean HBA1c was 9.0 ± 2.5 %.

Fasting plasma glucose less than 7.2 mmol/l was seen in 60.2 % of patients, 44.4 % had PPG levels less than 10 mmol/l, while only 22.2 % achieved HbA1c values less than 7 %.

### C peptide values

The mean fasting C-peptide was 1.44 ± 1.80 ng/ml. Fasting serum C-peptide levels were classified into 3 groups as being normal when serum C-peptide is between 0.9 to 3.0 ng/ml; as low when it is less than 0.9 ng/ml and high when the serum C-peptide level is greater than 3.0 ng/ml. Low fasting C-peptide levels were seen in 56.7 % of the patients, 27.8 % had normal levels, while 15.6 % had elevated levels. The mean postprandial C peptide was 4.0 ± 2.8 ng/ml. Change in C peptide was calculated as postprandial C peptide minus fasting C peptide. The mean change in C peptide was 2.64 ± 2.25 ng/ml.

Though the mean fasting and postprandial C peptide levels decreased with increasing duration of diabetes, it did not achieve statistical significance (FCP; F = 0.34, p = 0.71, PCP; F = 0.61, p = 0.55).

The patients who were on insulin had lower fasting and postprandial C peptide levels than those on either oral drugs alone or diet (p = 0.001, 0.003 respectively).

### Beta cell response

The mean fasting beta cell responsiveness (M0) was 1.32 ± 1.71 and postprandial beta cell responsiveness (M1) was 4.42 ± 4.62. Both fasting and postprandial beta cell responsiveness did not correlate significantly with the duration of diabetes (p = 0.17, 0.21 respectively). There were also no significant correlations between M0 and age and also between M1 and age (p = 0.103, 0.082 respectively). There was however significant correlation between M0 and HbA1c and between M1 and HbA1c (p = 0.007 and p < 0.001).

M0 also showed significant correlations with FPG while M1 correlated with PPG.

The patients were divided into three groups according to their tertiles of M0 and M1 and various clinical characteristics were compared across the three groups (Tables 1 and 2).

**Table 1:** Patients characteristics according to tertiles of M0

**Characteristic**	**Tertile1**	**Tertile2**	**Tertile3**	**p**
No (%)	33(36.7 %)	28(31.1 %)	29(32.2 %)	
Age(years)	54.1 ± 2.0	58.7 ± 1.7	59.9 ± 2.2	0.09
Duration of diabetes(years)	8.0 ± 1.3	6.9 ± 1.5	5.6 ± 0.9	0.35
Prevalence of oral drug use (%)	20.7	24.1	33.3	0.01*
Prevalence of insulin use (%)	13.8	8	0	0.01*
BMI(kg/m^2^)	26.4 ± 1.0	26.5 ± 1.0	30.0 ± 1.3	0.04*
FPG(mmol/l)	9.2 ± 0.7	6.7 ± 0.5	6.4 ± 0.5	0.01*
PPG(mmol/l)	13.4 ± 0.7	9.8 ± 0.7	9.7 ± 0.7	<0.001*
GLU change(mmol/l)	4.2 ± 0.5	4.0 ± 0.4	3.9 ± 0.6	0.92
HbA1c (%)	10.9 ± 0.4	8.1 ± 0.3	8.1 ± 0.4	<0.001*
FCP(ng/ml)	0.07 ± 0.02	0.79 ± 0.1	3.5 ± 0.3	<0.001*
PCP(ng/ml)	1.65 ± 0.38	4.08 ± 0.51	6.16 ± 0.29	<0.001*

Data are expressed as means ± S.E.

BMI; body mass index, FPG; Fasting plasma glucose, PPG; postprandial glucose, GLU change (PPG minus FPG), HbA1c; glycated haemoglobin, FCP; fasting C peptide, PCP; postprandial C peptide.

Comparisons between means made with ANOVA and with Chi-square for proportions.

*denotes significant values.

**Table 2:** Patients characteristics according to tertiles of M1

**Characteristic**	**Tertile1**	**Tertile2**	**Tertile3**	**p**
Age (years)	57.1 ± 2.1	53.7 ± 2.2	62.2 ± 1.4	0.009
Duration of diabetes(years)	8.3 ± 1.4	6.5 ± 1.2	5.5 ± 0.9	0.25
BMI (kg/m2)	26.5 ± 1.1	28.8 ± 1.2	27.5 ± 1.0	0.35
Prevalence oral drug use (%)	17	30.7	30.7	<0.001
Prevalence insulin use (%)	15.9	3.4	2.3	<0.001
FPG(mmol/l)	8.6 ± 0.6	7.8 ± 0.6	6.0 ± 0.6	0.01
PPG (mmol/l)	12.8 ± 0.8	11.7 ± 0.6	8.5 ± 3.4	<0.001
GLU change (mmol/l)	4.3 ± 0.5	5.1 ± 0.5	2.7 ± 0.3	<0.001
HbA1c (%)	10.0 ± 2.5	9.3 ± 0.4	7.8 ± 0.4	0.002
FCP (ng/ml)	1.40 ± 0.43	1.47 ± 0.31	1.52 ± 0.25	0.97
PCP (ng/ml)	1.40 ± 0.41	4.16 ± 0.43	6.31 ± 0.25	<0.01

Data are expressed as means ± S.E.

BMI; body mass index, FPG; Fasting plasma glucose, PPG; postprandial glucose, GLU change (PPG minus FPG), HbA1c; glycated haemoglobin, FCP; fasting C peptide, PCP; postprandial C peptide.

Comparisons between means made with ANOVA and with Chi-square for proportions.

*denotes significant values.

There were significant differences in diabetes treatment across the three groups. None of the patients in the first (highest) tertile of M0 was on insulin treatment, whereas 63.2 % of them were on diet alone. In the last tertile of M0, there were no patients on diet alone, while 66.7 % of them were being managed on insulin.

Also, mean glycated haemoglobin levels decreased across increasing tertiles of fasting beta cell responsiveness (p < 0.001) and also post prandial beta cell responsiveness (p = 0.002). The relationships between M0, M1 and other characteristics of the patients are as shown in Tables 1 and 2.

A comparison of the mean fasting C peptide, M0 and M1 in patients with good glycaemic control (HbA1c < 7 %) and those with poor glycaemic control (HbA1c ≥ 7 %) showed that those with poor glycaemic control had lower FCP, M0 and M1. However these differences were only statistically significant for M1 (p < 0.001) but did not achieve statistical significance for FCP and M0. (Table 3).

**Table 3:** Comparisons between patients according to glycaemic control

**Means**	**HbA1c < 7 %**	**HbA1c > 7 %**	**p**
FC peptide (ng/ml)	1.5	1.4	0.76
M0	1.8	1.2	0.67
M1	14.4	6.5	0.17

### Multiple linear regression with HbA1c as a dependent variable

In step-wise linear regression analysis using age, gender, duration of diabetes, M0, M1 as independent variables and HbA1c as the dependent variable, M0 and M1 emerged as significant predictors of glycated haemoglobin. With M0 as a variable, the R^2^ value was 0.149, while for M1, R^2^ was 0.207.

## Discussion

This was a cross-sectional study in type2 diabetes patients in which their beta cell function was assessed by testing their response to a standardized meal.

Low fasting C peptide levels was common in the patients occurring in over half of them, suggesting low beta cell reserve. This was seen despite the relatively short duration of diabetes in the patients (mean duration 6.7 yrs). This has been reported in similar studies demonstrating low beta cell reserve in African patients with type2 diabetes [8] .

There was a progressive decrease in both fasting and postprandial beta cell responsiveness with increasing duration of diabetes in the patients though unlike in other studies [7,13], this did not reach statistical significance. It is likely that this observation may be due to the different rates of beta cell deterioration in the patients. In addition, in our environment, a lot of patients with diabetes present to the clinic very late in their illness due to several factors such as ignorance, poor access to health facilities and patronage of alternative medical practitioners. Hence, a long period of hyperglycaemia and exposure of the beta cells to excessive glucose and the resultant glucotoxicity may result in earlier loss of beta cell function even in those with an apparently shorter duration of diabetes. Similarly in the study by Bakari, fasting plasma insulin did not correlate with duration of diabetes [9].

Generally there was poor glycaemic control in majority of the patients. In addition, M0 and M1 showed significant correlations with HbA1c and indeed were significant predictors of HbA1c in the regression model. Indeed further statistical analyses showed that among the three tertiles of both M0 and M1, HbA1c was lowest in those in the highest tertiles of both M0 and M1 though even in the patients in the highest tertile of M1, mean HbA1c was still high (7.8 %). Similarly patients who had poor fasting and postprandial beta cell responsiveness and fell into the lower tertiles of M0 and M1 also had worse glycaemic control in all parameters i.e FPG, PPG and HbA1c than those in the higher tertiles. This was also observed in the study by Shim et al [13] who reported that patients within the lowest tertile of postprandial C peptide levels had higher FPG, PPG and HbA1c levels. This suggests that the response of the beta cell is a significant determinant of glycaemic control in patients as also seen in studies by Shim et al [13] and Albarrak [7], in which both M1 and M0 were predictors of HbA1c.

However, when the patients were divided into two groups according to their glycaemic control those with poor glycaemic control had lower FCP, M0 and M1 than those with good glycaemic control though these differences were more significant for M1. Hence these differences were more marked with postprandial beta cell responsiveness which has been shown in other studies to be more predictive of glycaemic control than the fasting beta cell responsiveness [13].

Most of the patients who were on insulin were seen in the lowest tertiles of M1 and M0, and none of the patients in the highest tertiles was on insulin treatment. This is as expected as those on insulin are likely those patients who had been poorly controlled on oral agents, suggesting poor beta cell function. Similarly, Oli et al [11] reported that lower glucagon stimulated C peptide levels predicted sulphonyl urea failure and need for insulin treatment in Nigerian patients with type2 diabetes.

It was observed that a lot of the patients with poor beta cell function (lowest tertiles of M0 and M1) were managed with oral agents alone. Majority of them were on sulphonylureas which act by stimulating endogenous insulin release from the beta cells. Despite this, they still had poor beta cell stimulation from the meal and their drugs. It is possible that insulin treatment in this group would have resulted in better indices of glycaemic control in them.. The use of oral drugs in the patients with relatively poor beta cell responsiveness may have contributed to the high degree of poor glycaemic control.

One of the limitations of this study was the inability to collect multiple blood samples for the analysis of C peptide within the two hour sampling period, hence the need to use a simplified method for estimation of beta cell responsiveness. Though this equation has been used in a previous study [13], it is not known if more detailed sampling and C peptide modeling would yield similar results. In addition, antibody testing was not done to clearly differentiate between type1 and type2 diabetic patients and this may have affected the C peptide measurements as a few patients may have been improperly classified as having type2 diabetes.

## Conclusion

This study has shown that quite a significant proportion of our patients have low C peptide levels with poor beta cell response to meal stimulation. This suggests a rapid decline in beta cell function in them and that beta cell failure is a predominant feature in our patients with type2 diabetes. Late presentation for treatment with a long level of exposure of the beta cells to glucose from prolonged hyperglycaemia is likely to be a significant contributor to this decline in beta cell function. Fasting and postprandial beta cell responsiveness were significant determinants of blood glucose and glycated haemoglobin levels.

## Competing interests

The authors declare that they have no competing interests.

## Authors’ contributions

EY conceived and designed the study, collected the data, carried out the statistical analysis and wrote the draft of the manuscript. SC participated in the study design and data collection and also did major corrections in the manuscript. CU participated in data collection and final revision of the manuscript. All authors read and approved the final manuscript.

## Funding

The study was funded by personal financial contributions by each author. There was no external funding.

## Pre-publication history

The pre-publication history for this paper can be accessed here:

http://www.biomedcentral.com/1472-6823/12/11/prepub
